# Significance of Antigen and Epitope Specificity in Tuberculosis

**DOI:** 10.3389/fimmu.2014.00524

**Published:** 2014-10-23

**Authors:** Juraj Ivanyi, Tom H. M. Ottenhoff

**Affiliations:** ^1^Guy’s Hospital Campus of Kings College London, London, UK; ^2^Department of Infectious Diseases, Leiden University Medical Center, Leiden, Netherlands

**Keywords:** tuberculosis, antigens, bacterial, T-lymphocyte, epitopes, MHC restriction, vaccine adjuvant, TB diagnosis

Tuberculosis (TB) remains a major global health problem, because (i) diagnosis is usually made too late to avoid spread of infection to contacts; (ii) vaccination with bacillus Calmette–Guérin (BCG) does not prevent the most prevalent pulmonary disease; and (iii) defaulting from lengthy chemotherapy leads to an increase in drug resistant strains. The continued impact of human immunodeficiency virus (HIV) co-infections remains a major aggravating factor in TB resurgence. Intensive research on the specificity and function of immunological responses is of major importance since protective host defense is critically dependent on T cells, which selectively recognize only certain antigens and epitopes of the tubercle bacillus. Such knowledge is therefore necessary for designing a novel effective vaccine and better diagnostic tools. The specificity of the host immune response may also help to explain how the intracellular tubercle bacilli evade host resistance, probably by decoy pro-inflammatory actions of some of their antigens and/or immunomodulatory constituents, which lead to chronic infection and lung pathology.

The 12 articles in this Research Topic in the section Microbial Immunology of the journal Frontiers in Immunology review current knowledge, as well as gaps in our understanding of the mechanisms and functions of T and B cell recognition of antigens and constituent epitopes of *Mycobacterium tuberculosis* (Mtb). The abundant occurrence of major histocompatibility complex (MHC) class II-permissive epitopes in tubercle bacilli has important implications for the development of both subunit vaccines and diagnostic tests (1, 2). Moreover, an evolutionary interpretation has been that selection of Mtb strains carrying protective MHC-permissive epitopes could have extended the survival of infectious individuals and hence was advantageous for the protracted aerosol transmission of the pathogen (1).

Better understanding of the “antigenome” of Mtb has been advancing with the aid of new powerful strategies for the identification of antigens and its epitope determinants. These methods include bioinformatic approaches toward genome wide predictive algorithms for HLA binding and high-throughput tetramer generation (2). Hypothesis driven approaches or hypothesis free searches of the whole Mtb genome and functional screening algorithms led to the evaluation of candidate antigens, which are recognized well by T cells from latently infected individuals. They involve antigens expressed *in vivo*, encoded by the *DosR* regulon, resuscitation promoting factor (Rpf) proteins, and new HLA-class Ia or Ib (HLA-E) restricted Mtb epitopes, recognized by classical and non-classical CD8 T cells (3).

*Mycobacterium tuberculosis*-specific epitopes of immunodominant and HLA-permissive nature have been used extensively in IFNγ release assays (IGRAs). Several test kits can detect latent Mtb infection with better specificity than the tuberculin based skin test. However, these kits still need improving on their sensitivity and fail to distinguish active TB from latent infection. Moreover, biomarkers for predicting the risk of latent TB progressing into active TB are yet to be found (1, 3, 4). Further research on possible associations between epitope specificity and the phenotype of responding T cells could be an area of potential importance. Polyfunctional T cells have been associated with protective immunity on the grounds that the number of T cells producing IFNγ, IL-2, and/or tumor necrosis factor-α (TNFα) is correlated with vaccine induced protection in models of infectious diseases (4). However, the role of these cells is a subject of debate, since they are readily detectable also in patients with active or past TB. Although these cytokines are produced by several cell types [CD4, CD8, TCRγδ, mucosa-associated invariant T cells (MAIT), CD1-restricted T cells, and natural killer (NK) cells], it is significant that CD4 T cell depletion cannot be compensated with cell types other than CD4 T cells. Epitope-specific serum antibody levels in TB patients have been found to be influenced by the pulmonary bacterial load (associated with HLA-DR15), recent exposure to infection, and response to chemotherapy (5).

Identifying those antigenic determinants, which lead to host protection, is mandatory for designing more effective vaccination strategies. A recently failed vaccine trial in children employed Ag85A, which is highly immunogenic, but changes in its expression in infected cells could influence the susceptibility of infected cells to host immunity (6). Hence, there is a need to select suitable candidate antigens by more rigorous comparison of their protective capacity in animal vaccination models, before proceeding toward evaluation in human trials. In addition to antigen specificity, the success of a subunit vaccine may lie in its presentation, i.e., in the adjuvant formulation. To this effect, the fusion of antigens with interleukins, lipids, lipoproteins, and immune stimulatory peptides has been employed (7). Continued efforts to obtain better protection using recombinant strains of BCG engage over-expression of either Mtb-specific antigens (which had been lost during the attenuation process of BCG) or of some cytokines (IL-2, IL-12, IL-15, and GM-CSF) (8).

Although classical CD4 and CD8 T cells recognize peptide epitopes bound to MHC, molecules with different chemical structures could be of potential importance. Thus, T cells recognizing lipid antigens may contribute to natural host protection and might potentially be exploited for subunit based vaccination (9). Another structural aspect is the role of post-translational modifications of proteins identified using mass spectrometry-based proteomics (10). Recent attention to the proline–glutamic acid (PE) family of cell surface expressed proteins has been due to their immunomodulatory properties and possible evasion from host immunity by antigenic variation. Immunogenicity was attributed to the PE domain, while the specific epitopes were localized within the polymorphic GC-rich sequence (PGRS) domain. However, sensitization in human beings was found to be associated with BCG vaccination, rather than latent Mtb infection (11). Apparently, still other families of antigens need to be evaluated in the search for biomarkers, which could distinguish between stable protection and a tendency for recrudescence in latently infected populations and also for monitoring the efficacy of protection following prophylactic vaccination (12).

In conclusion, further research on Mtb antigen and epitope specificities seems mandatory for realizing the crucially important aims of both prophylactic and post exposure vaccination against TB. Advancing the knowledge of antigenic determinants is essential also for differentiating patients with active TB from latently infected healthy subjects. There is potential in the ambitious search for specific immunological biomarkers for predicting the reactivation of TB in populations, both without and with HIV infection. These endeavors will undoubtedly need to be combined with better knowledge of the functional phenotypes of the respective T cell subsets. Other potential avenues are the construction of fusion proteins with improved vaccine adjuvanticity (7) and the proposed construction of T cell receptor (TCR)-like ligands for immunotherapy (1). Future research may benefit also from advances in computer algorithm based analysis of Mtb epitopes and host cytokine signatures, as well as from reduced costs of DNA and RNA sequencing and synthetic peptide libraries.

## Conflict of Interest Statement

The authors declare that the research was conducted in the absence of any commercial or financial relationships that could be construed as a potential conflict of interest.

## References

[1] Ivanyi J. Function and potentials of *M. tuberculosis* epitopes. Front Immunol (2014) 5:107. 10.3389/fimmu.2014.00107 24715888 PMC3970012

[2] Lindestam Arlehamn CS Sette A. Definition of CD4 immunosignatures associated with MTB. Front Immunol (2014) 5:124. 10.3389/fimmu.2014.00124 24715893 PMC3970006

[3] Geluk A van Meijgaarden KE Joosten SA Commandeur S Ottenhoff THM. Innovative strategies to identify *M. tuberculosis* antigens and epitopes using genome-wide analyses. Front Immunol (2014) 5:256. 10.3389/fimmu.2014.00256 25009541 PMC4069478

[4] Prezzemolo T Guggino G La Manna MP Di Liberto D Dieli F Caccamo N. Functional signatures of human CD4 and CD8 T cell responses to *Mycobacterium tuberculosis*. Front Immunol (2014) 5:180. 10.3389/fimmu.2014.00180 24795723 PMC4001014

[5] Bothamley GH. Epitope-specific antibody levels in tuberculosis: biomarkers of protection, disease, and response to treatment. Front Immunol (2014) 5:243. 10.3389/fimmu.2014.00243 24917863 PMC4040437

[6] Huygen K. The immunodominant T-cell epitopes of the mycolyl-transferases of the antigen 85 complex of *M. tuberculosis*. Front Immunol (2014) 5:321. 10.3389/fimmu.2014.00321 25071781 PMC4089088

[7] Junqueira-Kipnis AP Marques Neto LM Kipnis A. Role of fused *Mycobacterium tuberculosis* immunogens and adjuvants in modern tuberculosis vaccines. Front Immunol (2014) 5:188. 10.3389/fimmu.2014.00188 24795730 PMC4005953

[8] da Costa AC Nogueira SV Kipnis A Junqueira-Kipnis AP. Recombinant BCG: innovations on an old vaccine. Scope of BCG strains and strategies to improve long-lasting memory. Front Immunol (2014) 5:152. 10.3389/fimmu.2014.00152 24778634 PMC3984997

[9] De Libero G Mori L. The T-cell response to lipid antigens of *Mycobacterium tuberculosis*. Front Immunol (2014) 5:219. 10.3389/fimmu.2014.00219 24904574 PMC4033098

[10] van Els CACM Corbière V Smits K van Gaans-van den Brink JAM Poelen MCM Mascart F Toward understanding the essence of post-translational modifications for the *Mycobacterium tuberculosis* immunoproteome. Front Immunol (2014) 5:361. 10.3389/fimmu.2014.00361 25157249 PMC4127798

[11] Cohen I Parada C Acosta-Gío E Espitia C. The PGRS domain from PE_PGRS33 of *Mycobacterium tuberculosis* is target of humoral immune response in mice and humans. Front Immunol (2014) 5:236. 10.3389/fimmu.2014.00236 24904584 PMC4033847

[12] Serra-Vidal M Latorre I Franken KLCM Díaz J de Souza-Galvão ML Casas I Immunogenicity of 60 novel latency-related antigens of *Mycobacterium tuberculosis*. Front Microbiol (2014) 5:517. 10.3389/fmicb.2014.00517 PMC4189613 25339944

